# Supplementary material for: Elevated MMP9 Expression—A Potential In Vitro Biomarker for COMPopathies
Source: Int J Mol Sci. 2025 Dec 15;26(24):12070. doi: 10.3390/ijms262412070 (PMC12732529; doi:10.3390/ijms262412070)
Supplement: Supplementary file 1 [file ijms-26-12070-s001.zip › ijms-4001366-supplementary.pdf]

Supplementary Table S1. KEGG pathway analysis of D469del COMP HT1080 cells.

Term	Adjusted p-value	Overlap	Genes
Pathways in cancer	1.17E-06	59/531	*CXCL8, LAMC2, GLI1, CCND2, CCND1, RASSF5, BDKRB2, BDKRB1, EDN1, GSTO2, WNT5B, DAPK1, MMP1, DCC, WNT5A, FOS, TRAF1, MMP9, TGFBR2, PLCB4, RARB, PLCB1, BIRC3, CSF1R, NOTCH3, LAMA5, EPAS1, LAMA1, LAMA3.*
AGE-RAGE signalling pathway in diabetic complications	7.63E-04	17/100	*EGR1, TGFB2, EDN1, SMAD3, CXCL8, F3, AGT, TGFBR2, VEGFA, MAPK11, IL6, PLCB4, CCND1, IL1B, PLCG2, BCL2, PLCB1.*
Cellular senescence	7.63E-04	22/156	*TGFB2, SMAD3, CXCL8, GADD45B, GADD45A, ITPR1, CDC25A, TGFBR2, HIPK2, MAPK11, IL6, RAD50, CCND2, CDK6, MRAS, CCND1, RASSF5, E2F1, CALM3, MYBL2, E2F5, CALM2.*
Apelin signalling pathway	8.66E-04	20/137	*NOTCH3, EGR1, SMAD3, JAG1, ITPR1, PLAT, ADCY1, GNG11, PIK3CG, APLN, MRAS, PLCB4, CCND1, GNG7, SPP1, CALM3, CCN2, PLCB1, PPARGC1A, CALM2.*
ECM-receptor interaction	0.001090706	15/88	*LAMA5, ITGA4, SDC4, ITGB4, LAMA1, ITGB3, LAMA3, LAMC2, THBS2, THBS1, COMP, FRAS1, SV2A, ITGA11, SPP1.*
Bladder cancer	0.001090706	10/41	*CXCL8, CCND1, MMP1, DAPK1, E2F1, THBS1, MMP9, FGFR3, VEGFA, HBEGF.*
PI3K-Akt signalling pathway	0.001258159	36/354	*CSF1R, LAMA5, ITGB4, LAMA1, ITGB3, LAMA3,*

			PDGFB, LPAR3, LAMC2, THBS2, THBS1, PIK3CG, COMP, CCND2, CCND1, CREB3L1, GNG7, PDGFC, SPP1, ITGA4, BDNF, GNG11, VEGFA, EFNA1, NR4A1, KITLG, IL6, CDK6, PPP2R2C.
Rap1 signalling pathway	0.003754453	24/210	*VAV3, FARP2, CSF1R, ITGB3, ITGB2, PDGFB, LPAR3, ADCY1, THBS1, VEGFA, EFNA1, MAPK11, KITLG, MRAS, PLCB4, RASSF5, PDGFC, CALM3, RAPGEF5, PLCB1, DRD2, FGFR4, CALM2, FGFR3.*
Focal adhesion	0.003754453	23/201	*VAV3, LAMA5, ITGA4, ITGB4, LAMA1, ITGB3, LAMA3, PDGFB, PARVA, LAMC2, THBS2, THBS1, VEGFA, COMP, CCND2, CCND1, PDGFC, ITGA11, SPP1, BCL2, FLNC, MYL9, BIRC3.*
Malaria	0.003754453	10/50	*COMP, TGFB2, IL6, CXCL8, LRP1, IL1B, ITGB2, PECAM1, THBS2, THBS1.*
Amoebiasis	0.003754453	15/102	*SERPINB3, SERPINB4, LAMA5, TGFB2, CXCL8, LAMA1, ITGB2, LAMA3, LAMC2, ADCY1, CXCL3, IL6, PLCB4, IL1B, PLCB1.*
Fluid shear stress and atherosclerosis	0.003789914	18/139	*GSTM3, EDN1, GSTM1, SDC4, GSTO2, ITGB3, PDGFB, PLAT, FOS, MMP9, VEGFA, MAPK11, CTSL, IL1B, BCL2, PECAM1, CALM3, CALM2.*
Parathyroid hormone synthesis, secretion and action	0.004192093	15/106,	*EGR1, PDE4D, ITPR1, GATA3, PTH1R, FOS, ADCY1, NR4A2, PLCB4, MMP15, CREB3L1, BCL2, PLCB1, CREB5, HBEGF.*

Transcriptional misregulation in cancer	0.004192093	22/192	*CSF1R, ARNT2, CXCL8, GADD45B, JUP, GADD45A, MMP3, PLAT, ETV1, TRAF1, MMP9, TGFBR2, BAIAP3, FUT8, IL6, HHEX, CCND2, NR4A3, IL2RB, NUPR1, SSX1, BIRC3.*
Lysosome	0.009653494	16/128	*ASAH1, SORT1, FUCA2, CLTC, LAPTM5, NAGA, ABCB9, LITAF, GM2A, NPC1, CTSL, LAMP3, ACP5, CTSH, DNASE2, ARSG.*
Small cell lung cancer	0.009653494	13/92	*LAMA5, GADD45B, LAMA1, GADD45A, LAMA3, LAMC2, TRAF1, CDK6, CCND1, E2F1, BCL2, RARB, BIRC3.*
Complement and coagulation cascades	0.014782949	12/85	*F8, SERPINB2, C1R, PROS1, ITGB2, BDKRB2, ITGAX, BDKRB1, PLAT, F3, CD55, F2RL2.*
TNF signalling pathway	0.01853741	14/112	*EDN1, JAG1, MMP3, LIF, TRAF1, FOS, CXCL3, MMP9, MAPK11, IL6, CREB3L1, IL1B, CREB5, BIRC3.*
Renin secretion	0.026485144	10/69	*EDN1, PLCB4, PDE1C, ITPR1, PDE3A, REN, CALM3, PLCB1, CALM2, AGT*
Rheumatoid arthritis	0.026485144	12/93	*TGFB2, IL6, CXCL8, CTSL, MMP1, IL1B, ITGB2, MMP3, ACP5, FOS, CXCL3, VEGFA.*
Human papillomavirus infection	0.026485144	29/331	*NOTCH3, LAMA5, ITGB4, LAMA1, ITGB3, LAMA3, LAMC2, THBS2, THBS1, OASL, COMP, CCND2, CCND1, CREB3L1, SPP1, E2F1, HES4, JAG1, WNT5B, ITGA4, WNT5A, MX1, TUBG2, VEGFA, CDK6, DLG3, PPP2R2C, ITGA11, CREB5.*
Vascular smooth muscle contraction	0.029268899	15/133	*EDN1, ITPR1, PLA2G3, PLA2G4A, ADCY1, ADRA1B, AGT, PLCB4,*

			KCNMB4, CALM3, PLCB1, MYL9, MYH10, CALM2, PRKG1.
Adrenergic signalling in cardiomyocytes	0.03336871	16/150	*ADCY1, ATP2B1, ADRA1B, PIK3CG, AGT, MAPK11, CACNB4, PLCB4, PPP2R2C, CREB3L1, BCL2, CALM3, SCN5A, PLCB1, CALM2, CREB5.*
Aldosterone synthesis and secretion	0.03336871	10/98	*NR4A2, NR4A1, PLCB4, CREB3L1, ITPR1, CALM3, ADCY1, ATP2B1, PLCB1, CALM2, AGT, CREB5.*
Inflammatory mediator regulation of TRP channels	0.03336871	10/98	*MAPK11, PLCB4, IL1B, ITPR1, PLCG2, BDKRB2, BDKRB1, PLA2G4A, CALM3, ADCY1, PLCB1, CALM2.*
Proteoglycans in cancer	0.03336871	20/205	*VAV3, TGFB2, SDC4, WNT5B, ITGB3, WNT5A, ITPR1, MIR21, MMP9, THBS1, VEGFA, MAPK11, MRAS, CCND1, CTSL, PLCG2, HCLS1, FLNC, EZR, HBEGF.*
cGMP-PKG signalling pathway	0.035959665	17/167	*ITPR1, ADCY1, ATP2B1, ADRA1B, PIK3CG, PLCB4, CREB3L1, PDE3A, BDKRB2, KCNMB4, CALM3, PDE5A, PLCB1, MYL9, CALM2, PRKG1, CREB5.*
Glutamatergic synapse	0.035959665	13/114	*SLC38A1, HOMER2, ITPR1, PLA2G4A, ADCY1, GNG11, GRK3, PLCB4, GNG7, SLC17A7, PLCB1, GLUL, SHANK3.*
Human cytomegalovirus infection	0.035959665	21/225	*CXCL8, ITGB3, ITPR1, CGAS, ADCY1, GNG11, VEGFA, MAPK11, IL6, PLCB4, CDK6, STING1, CCND1, CREB3L1, IL1B, GNG7, E2F1, CALM3, PLCB1, CALM2, CREB5.*
Pancreatic cancer	0.035959665	10/76	*TGFB2, SMAD3, CDK6, CCND1,*

			GADD45B, GADD45A, E2F1, TGFBR2, ARHGEF6, VEGFA.
Hepatocellular carcinoma	0.035959665	17/168	*GSTM3, TGFB2, SMAD3, GSTM1, WNT5B, GADD45B, GSTO2, GADD45A, TXNRD2, TXNRD1, WNT5A, TGFBR2, CDK6, CCND1, PLCG2, E2F1, DPF3.*
Cortisol synthesis and secretion	0.035964793	9/65	*NR4A1, PLCB4, CREB3L1, ITPR1, ADCY1, PLCB1, KCNK2, AGT, CREB5.*
Purine metabolism	0.035964793	14/129	*PDE1C, PDE4D, AK3, NME3, NME4, AK5, AMPD3, ADCY1, PDE3A, ENPP1, ENPP4, PDE5A, PDE7B, ADA.*
Relaxin signalling pathway	0.035964793	14/129	*EDN1, MMP1, FOS, ADCY1, GNG11, MMP9, TGFBR2, VEGFA, MAPK11, PLCB4, CREB3L1, GNG7, PLCB1, CREB5.*
Chagas disease	0.035964793	12/102	*MAPK11, TGFB2, IL6, CXCL8, PLCB4, PPP2R2C, IL1B, BDKRB2, FOS, ADCY1, PLCB1, TGFBR2.*
Regulation of actin cytoskeleton	0.043428031	20/218	*VAV3, ITGA4, ITGB4, ITGB3, ITGB2, PDGFB, MRAS, SCIN, SPATA13, PDGFC, ITGA11, BDKRB2, ITGAX, BDKRB1, EZR, FGFR4, MYL9, MYH10, FGFR3, ARHGEF6.*
Phospholipase D signalling pathway	0.045916844	15/148,	*CXCL8, PDGFB, PLA2G4A, LPAR3, ADCY1, PIK3CG, AGT, KITLG, MRAS, PLCB4, PDGFC, PLCG2, PLPP3, PLCB1, DGKI.*
Th17 cell differentiation	0.045916844	12/107	*MAPK11, IL6, SMAD3, IL1B, IL2RB, RORC, IL21R, AHR, GATA3, FOS, IL27RA, TGFBR2.*
IL-17 signalling pathway	0.045916844	9/94	*FOSL1, MAPK11, IL6, CXCL8, MMP1, IL1B, MMP3, FOS, CXCL3, MMP9, IL17RC.*

Hippo signalling pathway	0.046532841	16/163,	*TGFB2, SMAD3, WNT5B, WNT5A, ITGB2, AMOT, TGFBR2, BMP2, CCND2, CCND1, DLG3, PPP2R2C, CCN2, NF2, TEAD2, BIRC3.*
Kaposi sarcoma-associated herpesvirus infection	0.048009482	18/193	*CXCL8, PDGFB, ITPR1, FOS, CXCL3, GNG11, PIK3CG, VEGFA, PREX1, MAPK11, IL6, CDK6, CCND1, GNG7, PLCG2, E2F1, CALM3, CALM2.*

Supplementary Table S2. Mutagenesis primer list.

Mutation	Primer	Sequence (5’-3’)
p.C312Y	Forward	GCATCGGAGACGCCTACGATCCGGATGC
	Reverse	GCATCCGGATCGTAGGCGTCTCCGATGC
p.D385N	Forward	CCGCAACCAGGCCAACAACTGCCCTAG
	Reverse	CTAGGGCAGTTGTTGGCCTGGTTGCGG
p.G440R	Forward	GACCAGGATGGAGACCGACATCAGGACTCTC
	Reverse	GAGAGTCCTGATGTCGGTCTCCATCCTGGTC
p.D473H	Forward	GCGACGACGACGACCACAATGACGGAGTC
	Reverse	GACTCCGTCATTGTGGTCGTCGTCGTCGC
p.D511Y	Forward	GCCAGGACGACTTTGATGCATACAAGGTGGTAGA
	Reverse	TCTACCACCTTGTATGCATCAAAGTCGTCCTGGC

Supplementary Table S3. Antibody list.

Antigen	Host	Dilution	Company, product number
BiP	rabbit	1:1000	Cell Signalling Technologies, #3177
Calnexin	rabbit	1:1000	Enzo LifeSciences ADI-SPA 860
FLAG-HRP	mouse	1:1000	Sigma, A8592
phospho-eiF2a	rabbit	1:1000	Cell Signalling Technologies, #9721
Total eiF2a	rabbit	1:1000	Cell Signalling Technologies, #9722
GAPDH	mouse	1:40000	Merck Millipore, AB2302
GFP	rabbit	1:2000	Abcam, ab290
Mouse IgG	goat	1:2000	Dako, P0447
Rabbit IgG	goat	1:2000	Dako, P0448

Supplementary Table S4. qPCR primer list.

**Gene**	**Direction**	**Primer sequence (5'-3')**
GALNT18	Forward	GGTGGATGACAACAGCAGTAACG
	Reverse	GCTTGCTGTGACGCACGACTTT
MMP1	Forward	ATGAAGCAGCCCAGATGTGGA
	Reverse	TGGTCCACATCTGCTCTTGGCA
MMP9	Forward	GCCACTACTGTGCCTTTGAGTC
	Reverse	CCCTCAGAGAATCGCCAGTACT
SOX9	Forward	CCCATGTGGAAGGCAGATG
	Reverse	TTCTGAGAGGCACAGGTGACA
XBP1	Forward	GAAGCCAAGGGGAATGAAGT
	Reverse	CCAGAATGCCCAACAGGATA
HSPA5	Forward	GCTAATGCTTATGGCCTGGA
	Reverse	CGCTGGTCAAAGTCTTCTCC
18S	Forward	GGCCCTGTAATTGGAATGAGTC
	Reverse	CCAAGATCCAACTACGAGCTT

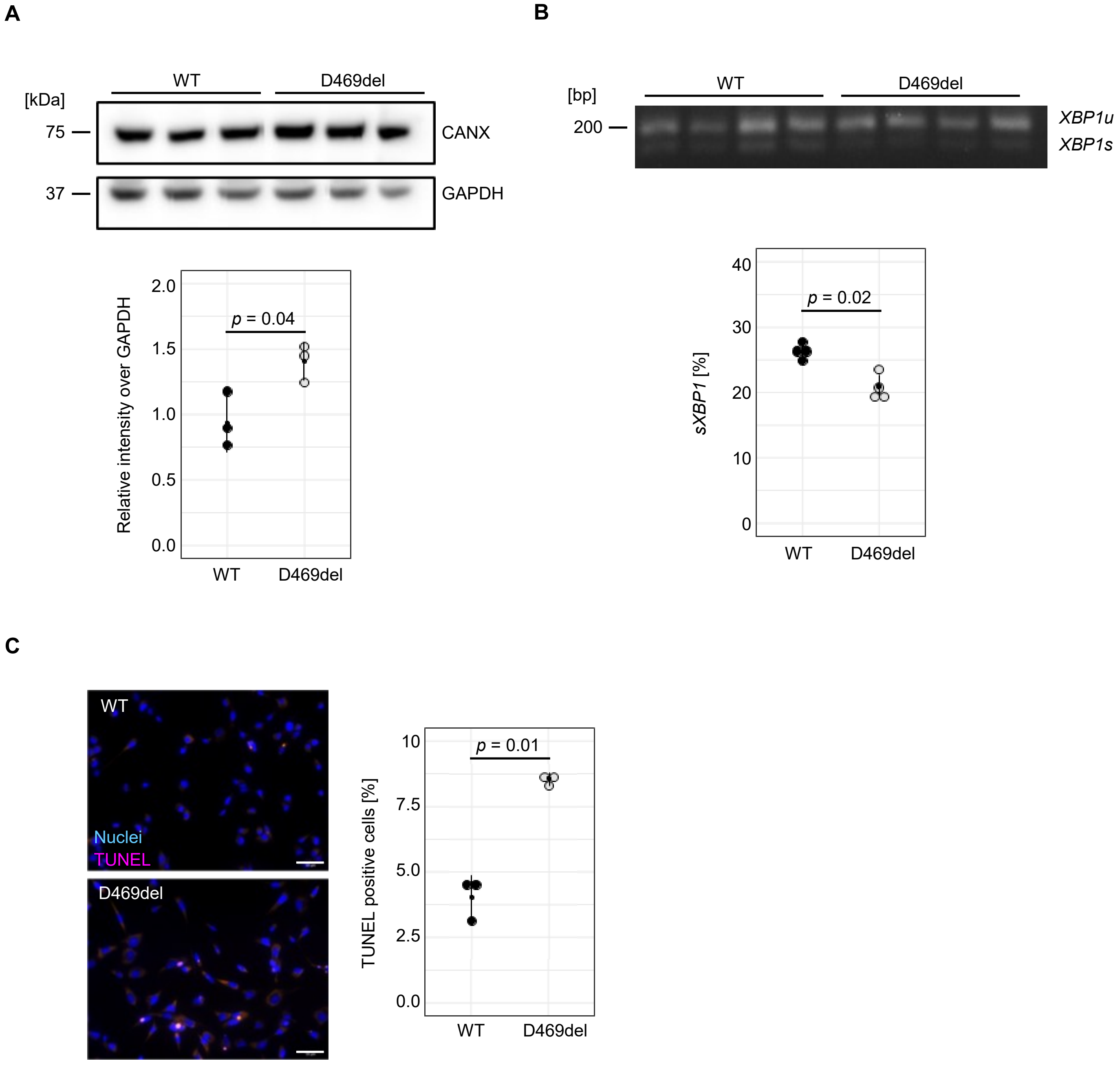

Supplementary Figure S1 – Analysis of calnexin levels, *XBP1* splicing and apoptosis in HT1080 cells overexpressing wild type and p.D469del COMP. (A) Representative western blot and quantification of calnexin in cell lysates from WT and p.D469del COMP-overexpressing HT1080 cells. GAPDH was used as a loading control. Mean and standard deviation of 3 experiments are shown. (B) *XBP1* splicing levels were examined by RT-PCR and agarose electrophoresis. Mean and standard deviation of 4 replicates are shown. *P*-values were determined using student's *t*-test. (C) Apoptosis is enhanced in p.D469del COMP compared to WT overexpressing cells. TUNEL assay was performed according to manufacturer's instructions. The number of TUNEL-positive cells was then normalised to the total number of cells (stained by DAPI), mean and standard deviation of 3 replicates are shown.

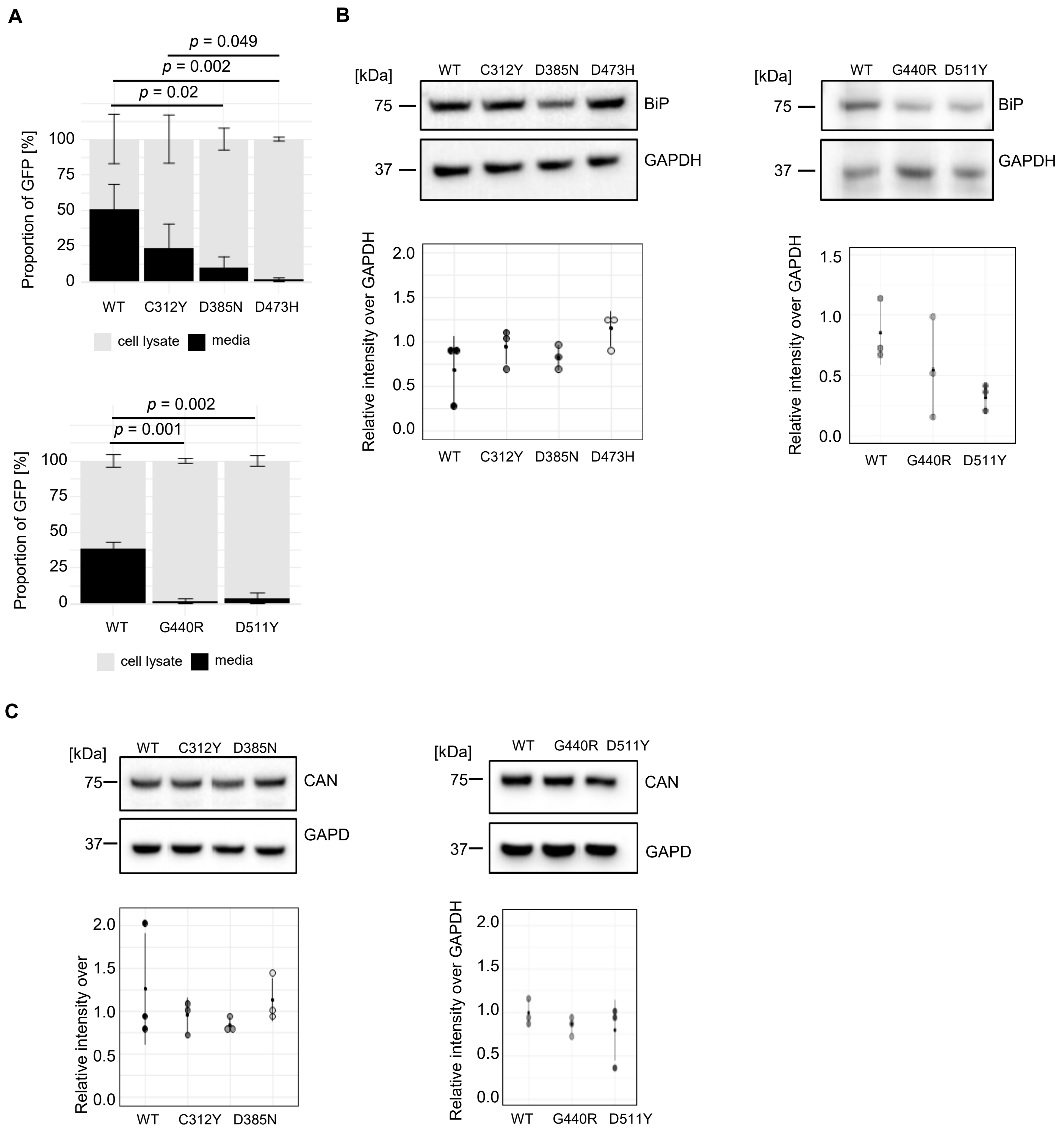

Supplementary Figure S2 – Intracellular COMP retention does not influence chaperone levels in cell models of COMPopathies. (A) Quantification of intracellular and extracellular COMP levels measured by western blotting in samples from CY312

(p.C312Y, MED), D385N (p.D385N, MED), D473H (p.D473H, PSACH), G440R (p.G440R, PSACH) and D511Y (p.D511Y, PSACH) compared to WT COMP cells. Error bars represent standard deviation of 3 experiments. (B) BiP levels and (C) calnexin levels were quantified by western blotting of cell lysates from WT, CY, DN and DY COMP cells. Mean and standard deviation of 3 experiments are shown. (A-C) GAPDH was used as loading control. ANOVA and Tukey's post-hoc test were used to determine *p*-values. *BiP was detected using the same membrane as Fig. 2A, therefore the same GAPDH loading control is shown.

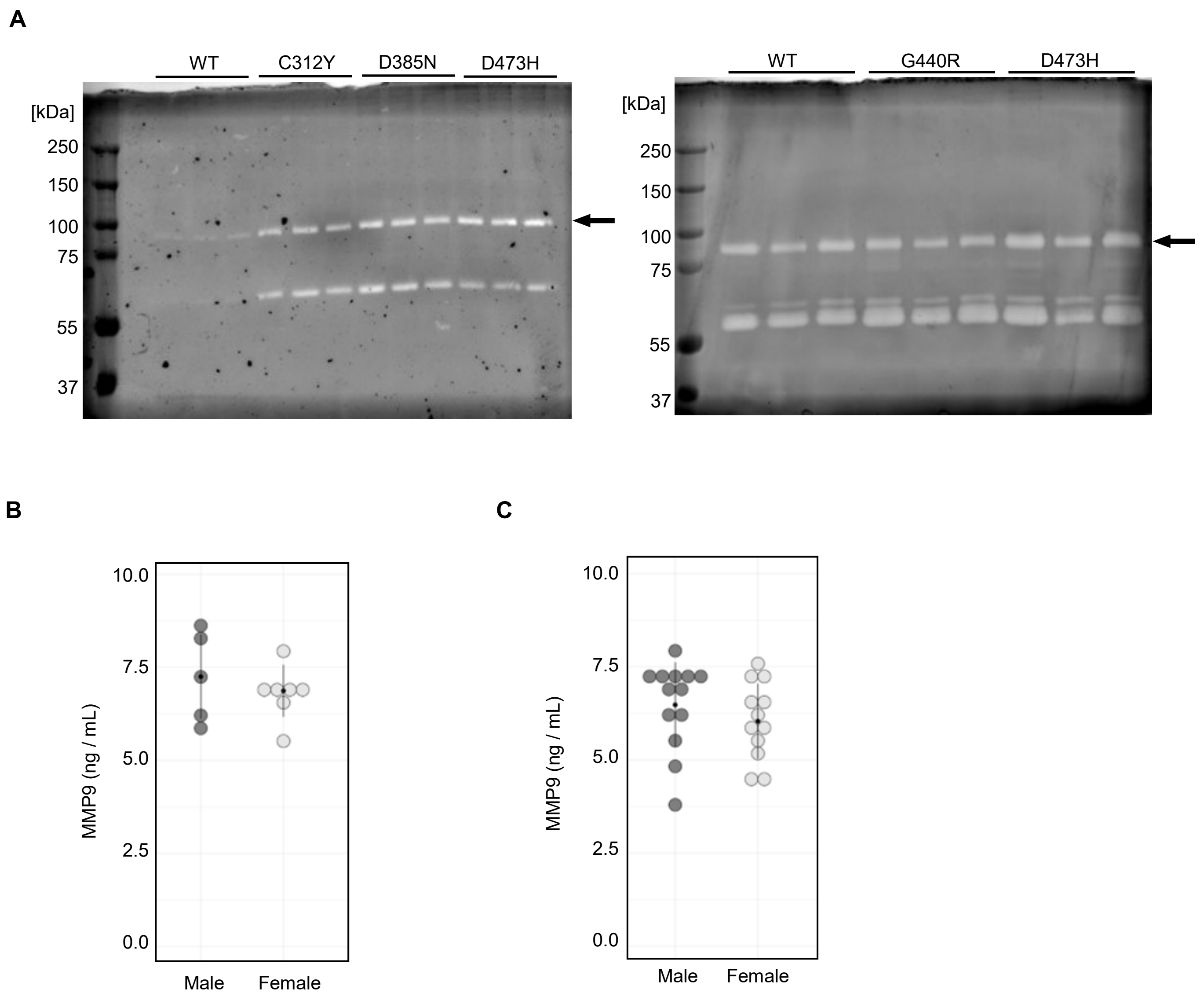

Supplementary Figure S3— (A) In-gel gelatin zymography was used to examine MMP9 and MMP2 activity in condi-tioned media of COMPopathy cell models. Arrow indicates molecular weight of active MMP9. (B, C) MMP9 serum levels do not differ between sexes of (B) wild type or (C) D469del COMP PSACH mice. Numbers indicate mice per group.

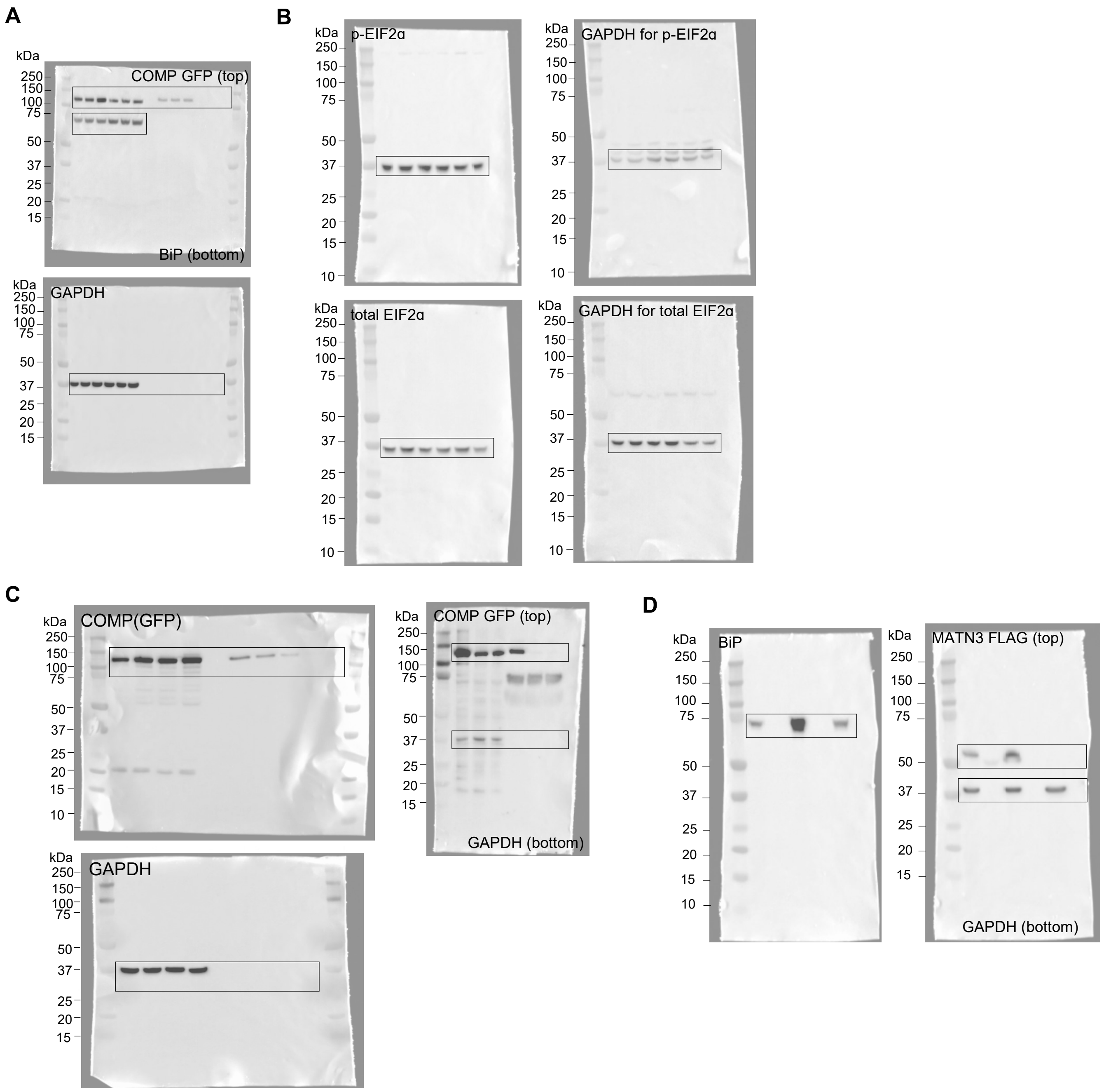

Supplementary Figure S4 – Full length western blots. (A) Blots shown in Figure 1A&B. (B) Blots shown in Figure 1C. (C) Blots shown in Figure 2C. (D) Blots shown in Figure 3A.

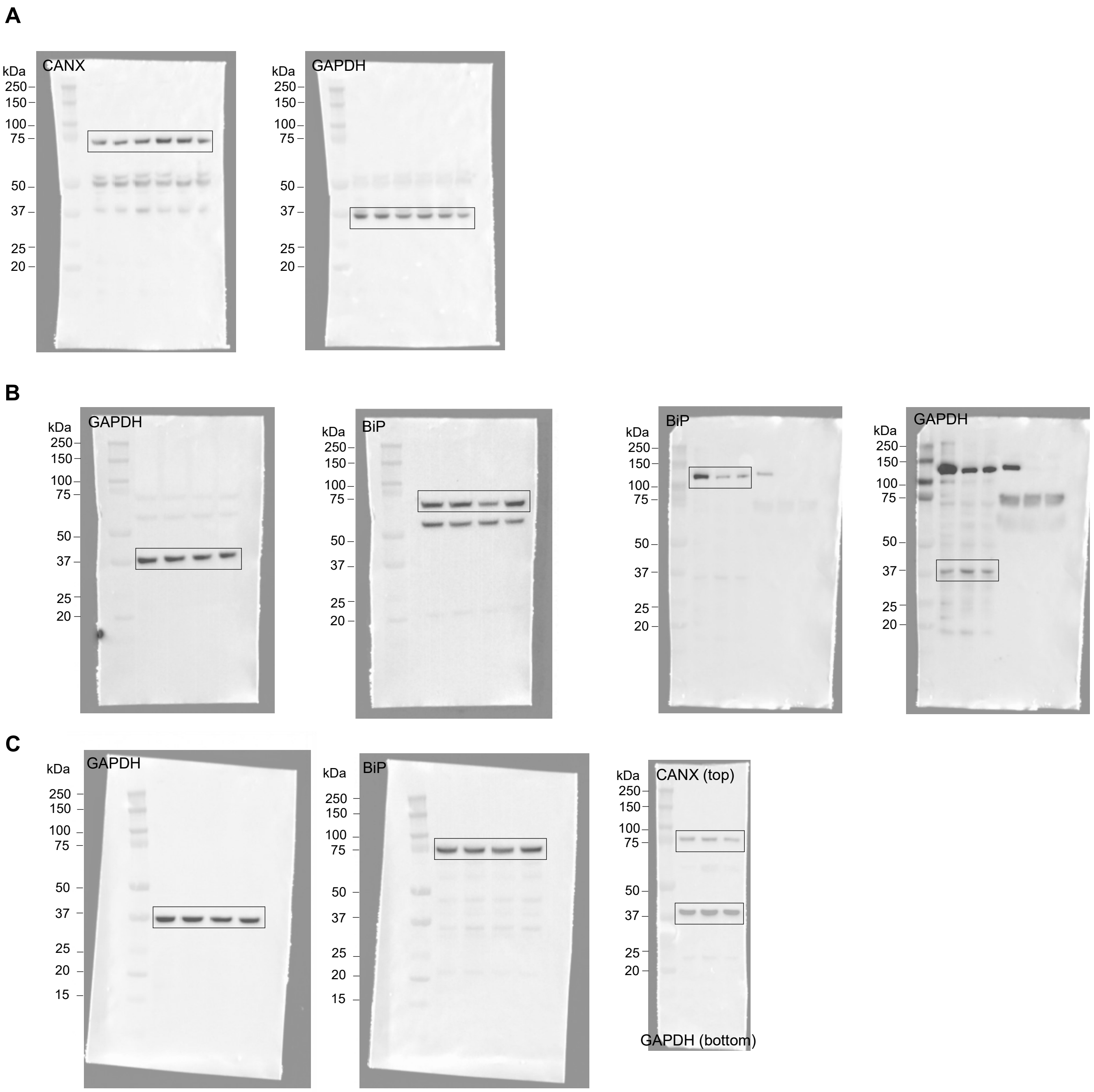

Supplementary Figure S5 – Full length western blots. (A) Blots shown in Supplementary Figure 1A. (B) Blots shown in Supplementary Figure 2B. (C) Blots shown in Supplementary Figure 2C.
